# Global Burden of Deep Neck Space Abscesses: Epidemiology, Challenges, and Outcomes

**DOI:** 10.3390/jpm16030146

**Published:** 2026-03-03

**Authors:** Antonino Maniaci, Francesco Chiari, Pierre Guarino, Luigi La Via, Mario Lentini, Salvatore Lavalle, Paolo Boscolo-Rizzo, Luigi Angelo Vaira, Jerome Rene Lechien

**Affiliations:** 1 Department of Medicine and Surgery, Enna Kore University, 94100 Enna, Italy; mario.lentini@unikore.it (M.L.); salvatore.lavalle@unikore.it (S.L.); 2 Study Group of the Young-Otolaryngologists (YO-IFOS), International Federations of Oto-Rhino-Laryngological Societies, 75000 Paris, France; pierreguarino@hotmail.com (P.G.); jerome.lechien@umons.ac.be (J.R.L.); 3 Department of Surgery, Mons School of Medicine, UMONS Research Institute for Health Sciences and Technology, University of Mons (UMons), 7000 Mons, Belgium; 4 Otolaryngology Head and Neck Unit, “Santo Spirito” Hospital, 65121 Pescara, Italy; francesco.chiari.med@gmail.com; 5 Department of Anaesthesia and Intensive Care, University Hospital Policlinico “G. Rodolico-San Marco”, 95123 Catania, Italy; luigilavia7@gmail.com; 6 Department of Medical, Surgical and Health Sciences, University of Trieste, 34149 Trieste, Italy; paolo.boscolorizzo@units.it; 7 Maxillofacial Surgery Operative Unit, Department of Medicine, Surgery and Pharmacy, University of Sassari, 07100 Sassari, Italy; lavaira@uniss.it; 8 Department of Otolaryngology-Head Neck Surgery, Elsan Polyclinic of Poitiers, 86000 Poitiers, France

**Keywords:** deep neck space abscesses, neck infections, suppurative abscesses, epidemiology, microbiology, surgical management, antimicrobial therapy, outcomes

## Abstract

**Background/Objectives**: Deep neck space abscesses (DNSAs), representing severe suppurative infections, continue to pose a significant global health challenge due to their morbidity, mortality, and evolving epidemiology. This review synthesizes existing knowledge regarding DNSA definitions, anatomic basis, epidemiological trends, microbiology, clinical presentation, diagnostic strategies, treatment paradigms, outcomes, health system challenges, and disparities to guide global efforts in DNSA prevention, management, and research. **Methods**: A structured narrative review was performed following SANRA guidelines. PubMed/MEDLINE and the Cochrane Library were searched from January 2000 to May 2025, retrieving 1102 records. After screening, 49 studies met the inclusion criteria. Data were extracted using standardized templates and synthesized thematically. **Results**: During the period 2004–2015, annual case increases were reported in a Finnish population-based retrospective cohort (n = 277), going from 14 to 24 subjects, and for a UK tertiary center retrospective series, going from 1 to 15 cases annually (2006–2015) (Pearson’s correlation, r = 0.9; *p* = 0.00019). The microbiological environment is mostly polymicrobial, composed of group streptococci and staphylococcus strains and anaerobes. Factors associated with poor outcomes include diabetes mellitus (adjusted hazard ratio of 10.7 [95% CI 6.0–19.1] in a retrospective, population-based cohort of 12,738 diabetic patients compared to 50,952 individuals without diabetes), immunosuppressed state, elderly age, and multispace involvement. Diagnosis relies on contrast-enhanced CT imaging (sensitivity > 90%), and treatment consists of early multidisciplinary intervention combining empiric broad-spectrum antibiotics with surgical drainage in 60–97% of cases. Mortality ranges from 1.6% to 7.6%, with higher rates in cases complicated by mediastinitis (up to 40%). **Conclusions**: DNSAs demonstrate a clear upward incidence trend across high-income and resource-limited settings. Establishing standardized DNSA registries, validating risk-stratification tools, reinforcing antimicrobial stewardship to address rising resistance, and implementing early detection protocols in primary care remain critical priorities. While emerging technologies, including rapid molecular diagnostics and AI-based decision support, represent promising research directions, current DNSA management relies fundamentally on conventional clinical assessment, prompt imaging, and coordinated multidisciplinary care.

## 1. Introduction

Deep neck space abscesses (DNSAs), representing severe suppurative infections within the potential fascial planes of the head and neck, underline a significant global health challenge due to their morbidity, mortality, and evolving epidemiology. Characterized by pus accumulation in anatomically distinct deep cervical compartments—such as the parapharyngeal, retropharyngeal, submandibular, and masticator spaces—these abscesses often originate from odontogenic, pharyngotonsillar, otogenic, or sinonasal sources [1,2,3,4]. The intricate fascial architecture facilitates rapid downward dissemination into the mediastinum or upward extension into adjacent cranial spaces, thereby increasing the risk of catastrophic complications, including airway compromise, descending necrotizing mediastinitis, jugular venous thrombosis, and intracranial spread [1,3,5]. Historically, the introduction of broad-spectrum antibiotics resulted in a marked decline in DNSA incidence; however, recent epidemiological evidence from both high-income (HICs) and low- and middle-income countries (LMICs) signals a resurgence. Recent population-based studies from Europe have documented significant upward trends in DNSA incidence over the past two decades [6]. Moreover, LMICs—particularly in South Asia—continue to bear a disproportionate DNSA burden, with socioeconomic determinants including limited healthcare access, lower education levels, and rural residence contributing to delayed presentations and increased severity [7].

DNSAs are commonly polymicrobial, reflecting the flora of the oral cavity and upper respiratory tract, and notably include aerobic Streptococcus and Staphylococcus species as well as anaerobic organisms—microbial profiles that carry implications for empiric antimicrobial regimens amid rising antimicrobial resistance concerns [8,9,10]. Vulnerabilities are magnified among children, the elderly, and individuals with comorbidities such as diabetes mellitus, immunosuppressive conditions, or renal failure, which have been repeatedly identified as predictors of severity, intensive care unit (ICU) admission, increased complications, and mortality [6,11,12].

Given these multifactorial drivers and outcomes, a globally contextualized, narrative synthesis of DNSA burden, management, and outcomes is urgently needed. This review aims to synthesize existing knowledge regarding DNSA definitions, anatomic basis, epidemiological trends, microbiology, clinical presentation, diagnostic strategies, treatment paradigms, outcomes, health system challenges, disparities, and evidence-based recommendations—thereby equipping clinicians, researchers, and policy-makers with a robust framework to guide global efforts in DNSA prevention, management, and research.

Given the heterogeneity in presentation, microbiological spectrum, and patient comorbidities, DNSAs represent a complex infectious disease model. Current management relies primarily on conventional clinical evaluation, imaging, and surgical decision-making.

## 2. Materials and Methods

### 2.1. Study Design

We conducted a structured narrative review following SANRA (Scale for the Assessment of Narrative Review Articles) guidelines to ensure methodological transparency. This approach was selected due to the heterogeneity in study designs, settings, and outcome measures across the DNSA literature, which precluded formal meta-analysis. Unlike systematic reviews, narrative reviews synthesize literature thematically rather than through standardized pooling of effect estimates; however, a structured search strategy enhances reproducibility.

### 2.2. Literature Identification and Screening

A structured search was conducted across PubMed/MEDLINE and the Cochrane Library from 1 January 2000 to 31 May 2025, retrieving 1102 records. After duplicate removal (n = 245), 857 unique records underwent title and abstract screening by two reviewers (A.M. and F.C.) using predefined eligibility criteria. Discrepancies were resolved through discussion with a third reviewer (J.R.L.). After title/abstract screening, 162 articles remained for full-text review. Excluded articles (n = 114) were single case or small case series (<5 patients), editorials and comments without original data, nontranslated non-English publications, anatomical studies without clinical outcomes, as well as animal and in vitro studies. The narrative synthesis comprised a total of 49 included studies. A flow chart of the screening process appears in Supplementary Figure S1.

### 2.3. Search Strategy

The search strategy combined Medical Subject Headings (MeSH) and free-text terms and was as follows: (“deep neck space abscess” [Title/Abstract] OR “deep neck infection” [Title/Abstract] OR “retropharyngeal abscess” [MeSH] OR “parapharyngeal abscess” [Title/Abstract] OR “submandibular abscess” [Title/Abstract]) AND (“epidemiology” [MeSH] OR “microbiology” [MeSH] OR “management” [Title/Abstract] OR “outcome” [Title/Abstract] OR “mortality” [MeSH] OR “treatment” [MeSH]) AND (“1 January 2000” [Date-Publication]: “31 May 2025” [Date-Publication]).

The Cochrane Library strategy was adapted using Cochrane-specific syntax with equivalent terms.

The search was last updated on 31 May 2025. A confirmatory search conducted on 15 November 2025 identified no additional eligible studies meeting the inclusion criteria.

Inclusion criteria were as follows:

- Study designs: Randomized controlled trials, prospective/retrospective cohort studies, case series ≥ 5 patients, systematic reviews, and meta-analyses;
- Population: All ages with deep neck space abscesses;
- Outcomes: Epidemiology, microbiology, clinical presentation, diagnostics, management, or outcomes;
- Language: English or with English translation available.

We excluded single case reports with fewer than five patients, animal studies, anatomical studies without clinical correlation, and opinion pieces lacking systematic review methodology. Complete search strings for both databases are provided in Supplementary Material Figure S1.

### 2.4. Data Extraction and Quality Assessment

Data were extracted by two reviewers (A.M. and F.C.) using a standard form. Variables assessed included the following: study (author, year, country, design, setting, sample size), population (age and sex), clinical data (abscess location, etiology, symptoms, complications), microbiology (organisms isolated and their resistance patterns), diagnostics (imaging modality), treatment management (antibiotic regimens used, surgical approach implemented if required and timing), and outcome values (length of stay in days, need for ICU admission, mortality). Differences were resolved by discussion with a third reviewer (J.R.L.). Quality was assessed using the Newcastle–Ottawa scale (for cohort studies) and modified criteria for case series, which included clarity of case definition, consecutive enrollment, complete outcome reporting, and adequacy of follow-up. Quality appraisal informed the weight assigned to each study in our synthesis but was not employed for exclusion, as per narrative review methodology. Where specific outcomes were not reported in studies, data were recorded as “not reported” rather than assumed.

### 2.5. Data Synthesis

Due to marked heterogeneity in study populations, settings, definitions, and outcomes, it was not possible to carry out a formal meta-analysis. We completed a structured thematically arranged narrative synthesis, including epidemiology, etiology, and the risk factors; microbiology and pathophysiology; clinical presentation and diagnostics; disease management strategy, including interventions, psychosocial care, service knowledge gaps, or silos; burden of diseases across the globe; healthcare system barriers; and prevention strategies.

Evidence for each theme was synthesized descriptively with attention to consistency or variation across studies and gaps in the literature. Where the consensus between studies was achieved, we provided ranges instead of pooled estimates. There were also greater interpretative weights given to evidence from higher-quality and larger studies, but all eligible studies fed into the synthesis. Statistical data reported in the results were verified against primary sources.

### 2.6. Limitations of Methodology

This narrative structured review also has some limitations. Limiting articles to those in English-language publications could inadvertently eliminate pertinent regional analyses. Publication bias probably favors the most serious patients and positive results. The lack of uniform definitions, diagnostic criteria, and outcome measures for DNSAs hinders direct comparisons between studies. LMIC statistics are mainly collected from the data of only one tertiary unit, with implications for generalizability. Research spanning 25 years may not reflect current practice, especially regarding antimicrobial resistance. This review is in narrative synthesis format, so no pooled effect estimates can be reported. These limitations influence the interpretation of findings and recommendations for future research.

## 3. Findings

This section assesses findings from 49 studies [3,4,5,6,7,8,9,10,11,12,13,14,15,16,17,18,19,20,21,22,23,24,25,26,27,28,29,30,31,32,33,34,35,36,37,38,39,40,41,42,43,44,45,46,47,48,49,50,51] in a synthesized narrative, thematically arranged around epidemiology, microbiology, clinical presentation, diagnostics, management, outcomes, healthcare system challenges, and strategies for mitigation. Epidemiological and clinical data have been confirmed with primary sources, and all figures reference their original studies to ensure transparency.

### 3.1. Epidemiology

#### 3.1.1. Global Incidence and Prevalence

**Figure 1:**
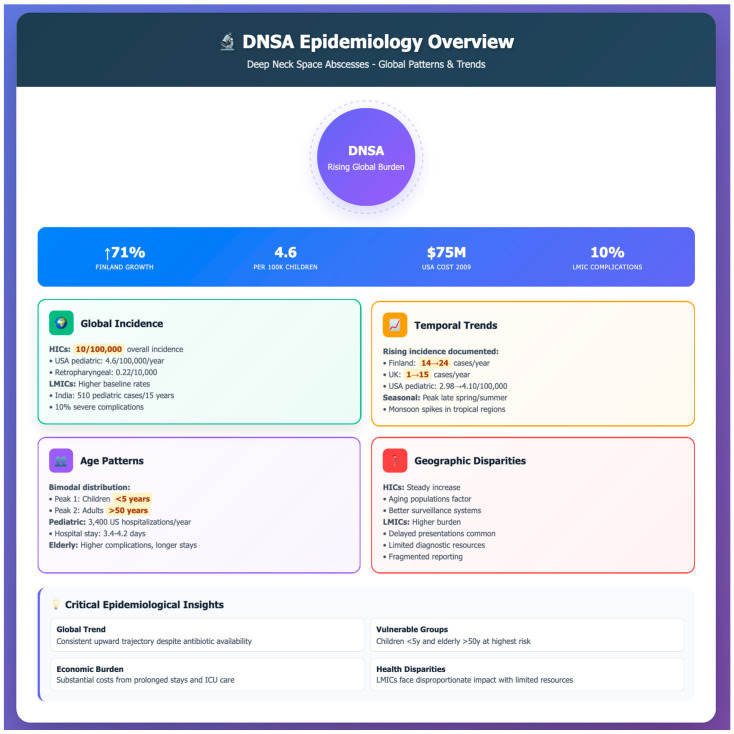
This figure presents a schematic overview synthesizing epidemiological patterns of deep neck space abscesses as reported across included studies. The diagram organizes key themes, including geographic distribution, age-specific patterns, temporal trends, and healthcare setting variations. While individual data points referenced within the figure (e.g., incidence rates, demographic distributions) are derived from cited primary studies [6,7,13,14,18], the overall organizational framework and visual layout represent an author-generated conceptual synthesis designed to facilitate reader comprehension of complex, heterogeneous epidemiological data. This figure does not present original pooled analyses or meta-analytic estimates.

Recent epidemiological studies illustrate marked geographic and socioeconomic disparities in the incidence and prevalence of DNSAs. In HICs, data from the United States suggest that there are approximately 3400 pediatric hospitalizations annually, with an incidence of 4.6 per 100,000 children per year [13], while overall DNSA prevalence hovers around 10 per 100,000 inhabitants, with specific rates of 0.22 and 0.14 per 10,000 for retropharyngeal and parapharyngeal abscesses, respectively [6]. Another U.S. study estimated a rate of 1.37 per 10,000 deep space neck infections in 2009 [14], underscoring a relatively steady yet non-negligible disease burden. In contrast, LMICs confront a higher and more variable incidence, often coupled with delayed presentation and limited healthcare infrastructure, though population-based epidemiological reporting remains fragmented. Tertiary center reports from India have documented sizable pediatric cohorts—for example, 510 children under five treated over 15 years—highlighting substantial institutional caseloads [7]. Despite the lack of robust national incidence data, hospital series indicate persistently elevated disease frequency and complication rates, including 10% incidence of severe outcomes in resource-limited settings [7,15,16,17] (Figure 1).

Age-specific incidence patterns reveal that pediatric populations—especially children under five—bear a disproportionately high burden. USA pediatric data consistently demonstrate an incidence of ~4.6 per 100,000, with retropharyngeal abscess rates increasing from 2.98 to 4.10 per 100,000 in those under 20 years between 2003 and 2012 [18]. In a retrospective case–control study from Taiwan comparing 56 diabetic and 87 non-diabetic DNSA patients treated at a single tertiary center (1997–2002), Huang et al. demonstrated distinct microbiological profiles: Klebsiella pneumoniae predominated in diabetics (56.1%, 32/57 isolates) compared to Streptococcus viridans in non-diabetics (43.7%, 38/87 isolates), with higher abscess formation rates in the diabetic group (89.3% [50/56] vs. 71.3% [62/87], *p* = 0.014, chi-square test) [16]. Among adults, prevalence rates approximate those of the general population in HICs, though distinct age-related complications emerge. In elderly cohorts, comorbidities such as diabetes and immunosenescence amplify risks and prolonged hospital stay, yet epidemiological estimates remain underreported in this population.

#### 3.1.2. Temporal and Geographic Trends

Population-based analyses over recent decades reveal a steady increase in DNSA incidence across multiple regions, particularly within HICs. In a population-based retrospective cohort study from Finland, Velhonoja et al. analyzed 277 consecutive DNSA patients admitted to a university hospital between 2004 and 2015, observing an increase in annual admissions from 14 to 24 cases over the study period [6]. Odontogenic etiology predominated (124/277, 44.8%), and ICU admission was required in 66 patients (23.8%). Patients with comorbidities demonstrated higher rates of ICU admission, longer ICU stays, and more frequent repeat surgeries, though specific comparative statistics were not reported.

In a nationwide population-based cohort study using Taiwan’s National Health Insurance Research Database, Chang et al. compared 12,738 patients with Type 1 diabetes mellitus to 50,952 age- and sex-matched controls without diabetes (1:4 ratio) over a mean follow-up of 7.2 years [17]. Type 1 diabetes was associated with significantly increased DNSA risk (adjusted hazard ratio 10.71; 95% CI 6.02–19.05; *p* < 0.001), adjusted for age, sex, urbanization, income, and comorbidities. Among patients who developed DNSA, those with diabetes experienced prolonged hospitalization compared to non-diabetics (mean 9.0 vs. 4.1 days, *p* < 0.001, Student’s *t*-test).

Seasonal variation is also noted, with higher incidence reported during late spring and summer months, consistent with immunological and environmental factors [19]. These increasing rates in HICs might be associated with aging populations, higher prevalence of comorbidities, and possibly shifts in microbial virulence [6].

In LMICs, geographic variability is pronounced, with studies indicating both higher baseline incidence and more severe disease presentations. Institutional data from India, for instance, report a sustained annual caseload with up to 10% of cases manifesting significant complications due to delayed presentation and constrained access to advanced diagnostics [7,20]. Although national registry data are rare, individual centers across Africa and Southeast Asia have reported similar high local incidence and seasonal spikes correlated with monsoon-related respiratory tract infections [21,22,23].

##### Geography and Formula Geographic Prevalence

Epidemiological patterns and microbiological profiles vary widely by geographic area and economic status, with significant implications for empiric treatment regimens and broadly based public health interventions.

In Europe, Northern European countries have described well-defined trends using population-based registries. The Finnish population-based study of Velhonoja et al. showed an increase in annual admissions from 14 to 24 cases over the period of 2004–2015 [6]. Equivalent increases have also been seen in the United Kingdom [32,37] and Western Europe. In pharyngotonsillar infections, microbiological profile is still dominated by Streptococcus pyogenes and Streptococcus anginosus groups in European settings, whereas oral streptococci and anaerobes (Prevotella, Fusobacterium, Peptostreptococcus) play a dominant role for odontogenic origin [8,9]. The prevalence of MRSA in European DNSA series is still relatively low (5–12%) compared to North American data [10].

In North America, the United States and Canada have experienced similar increases in DNSA rates with specific rises among diabetics and immunocompromised patients [13,14,18]. One of the unique aspects of North American epidemiology is a larger proportion of community-associated MRSA (caMRSA), which can contribute between 12 and 25% to staphylococcal isolates among some DNSA series [10]. This trend requires coverage of empiric MRSA (vancomycin, clindamycin, or linezolid) in high community MRSA areas. Adult populations are largely odontogenic-based, reflecting differences in access to dental care, especially among the uninsured and underinsured [13,21].

Epidemiological profiles of STHs are heterogeneous across Asia and vary according to economic development and the healthcare system. High-income East Asian countries (Japan, South Korea, Taiwan) present epidemiological patterns close to those in the West [17,23,24].

Takahashi et al. conducted a nationwide retrospective survey (2018–2023) in 39 Japanese pediatric hospitals of cervical abscess-forming infections before, during, and after COVID-19 pandemic restrictions [23]. For a total of 207 cases (96 superficial, 111 deep neck abscesses), monthly incidence rates (mean ± SD cases per institution) were significantly higher in the post-pandemic period than in the pre-pandemic one: deep from 0.50 ± 0.72 (pre-COVID) to 0.60 ± 0.78 (mid-COVID) to 1.67 ± 1.11 cases/month (post-COVID), with statistically produced significant increase recorded between them (*p* < 0.05, ANOVA with post hoc testing). This trend was connected to decreased population immunity associated with reduced pathogen exposure during pandemic suppression.

In contrast, DNSA in South and Southeast Asian countries, such as India, poses a heavy disease burden marked by more frequent cases of the condition, later presentations, and greater severity [7,20]. The reasons are due to lack of access to dental care, lack of early recourse to healthcare because of cost barriers, higher rates of uncontrolled diabetes, and deficient capacity in primary care. Microbiologically, there is also a higher amount of Klebsiella pneumoniae reported in South Asian series, especially in those patients who are diabetics, indicating a unique regional pattern with relevance for the choice of empiric antibiotic therapy [16]. The report of Huang et al. on the Taiwanese case–control study reported that the prevailing organism was Klebsiella pneumoniae in diabetics (32/57 isolates, 56.1%) as opposed to Streptococcus viridans in non-diabetics (43/87 isolates, 43.7%) [16]. From Asian centers, a few studies have reported extended-spectrum beta-lactamase (ESBL)-producing Gram-negative organisms, which are representative of the large-scale trends of antimicrobial resistance seen across the region [10].

Data from Sub-Saharan Africa are scarce and predominantly based on single-center retrospective series in South Africa, Nigeria, and Ethiopia [22,45]. The present evidence indicates a high DNSA burden, which is mainly odontogenic-induced due to restricted access to dental care. With delayed presentations, increased incidence of complications (mediastinitis; airway compromise and death are more likely to rapidly occur). HIV co-infection is a major comorbidity that can present atypically and with increased mortality. The available literature in Latin America, mainly from Brazil and Mexico, has reported the odontogenic origin as being most common with comparable microbiological profiles to studies conducted in Europe and North America [11,28]. Healthcare access inequities between urban and rural populations result in delayed presentations among the underserved. Comparable data for Australia, reported by Asairinachan et al., demonstrated that MRSA was recovered in 12% (15/127) of culture-positive cases, and ESBL-producing Gram-negative bacteria were isolated in 8% (10/127), patterns like those seen in Western European ICUs [10]. Australian Indigenous people are disproportionately subjected to the burden of DNSA due to increased prevalence of diabetes and limited access to healthcare [30].

##### Low- and Middle- Versus High-Income Countries—Comparative Analysis

When compared systematically, between LMICs and HICs, similar inequalities are found across various sectors. The prevalence data are, however, constrained by inconsistent surveillance, and the incidence data indicate a higher DNSA burden in LMICs due to poorer dental care infrastructure, deferral of healthcare seeking, and increased levels of uncontrolled diabetes [7,22]. In terms of etiology, odontogenic causes are more prevalent in LMICs (60–80%) compared to HICs (40–60%), with differences in availability and allocation of oral care [7,22]. Microbiological differences are clinically significant. Although streptococcus and oral anaerobes remain the most common pathogens globally, Gram-negatives are more commonly reported from LMICs (especially K. pneumoniae among diabetic patients) [16]. MRSA distribution is regional rather than solely income-based, with the highest rates in North America and some areas of Asia [10]. A similar line of thought was raised in the Romanian series by Bandol and co-workers, who reported 38% (34/89) of penicillin-resistant Staphylococcus aureus [8], indicating the spread of resistance in Eastern European areas. Clinical outcomes differ substantially. Recent HIC rates of mortality are between 1.6% (a Finnish cohort, with 4/277 patients) [6] and 7.5% (an UK series, 4/53 patients) [32], whereas LMIC reports have cited higher complication rates and death prevalence in direct relation to delay in presentation, advancement stage of the disease, limited intensive care resources or availability, and a restricted surgical resource pool [7,22,46] (Table 1).

### 3.2. Etiology and Risk Factors

#### 3.2.1. Primary Sources of Infection

The etiology of DNSAs is different according to the age and region in which patients live, leading the source of infection to be the main factor causing it, and this will directly affect the type of microorganisms isolated, the anatomical site affected, and how to treat them. Odontogenic cause is the most common etiology in adults, with 40–70% of all cases across reported series [6,9,22,23]. Infections generally arise from mandibular molars, especially second and third molars, such as periapical abscesses, periodontal disease, and post-extraction problems. The submandibular, sublingual, and masticator spaces are the most involved, with Ludwig’s angina being the classic bilateral infection of the submandibular space. Odontogenic DNSAs are commonly polymicrobial, consisting of oral streptococci (Streptococcus viridans group, Streptococcus anginosus group), anaerobes (Prevotella, Fusobacterium, Peptostreptococcus, Bacteroides), and occasionally Staphylococcus species [8,9]. This microbiological profile demands antibiotic therapy directed at both aerobic streptococci and anaerobes, e.g., ampicillin–sulbactam or clindamycin. Source control with dental extraction or root canal is mandatory and should ideally be carried out at the time of primary surgical drainage, to minimize recurrence risk [2,6]. Pharyngotonsillar sites represent the second most common origin globally and are the main cause in children, representing 20–40% of the cases [13,14,22]. The parapharyngeal and retropharyngeal spaces may be involved in acute tonsillitis, peritonsillar abscess, and adenoid infections. Suppurative lymphadenitis of the retropharyngeal lymph nodes (nodes of Rouvière) after URI is especially common in children younger than 5 years due to involution of these nodes with advancing age (by 5–6 years) [14,18]. Bacteria causing pharyngotonsillar DNSAs are mostly group A Streptococcus (Streptococcus pyogenes), Staphylococcus aureus, and respiratory anaerobes. Fusobacterium necrophorum is particularly linked with the Lemierre syndrome, which involves thrombophlebitis of the internal jugular vein and septic emboli [18]. It is important for empiric therapy to cover Gram-positive cocci and anaerobes, and penicillin-based combinations with beta-lactamase inhibitors or clindamycin are recommended first-line treatments. Tonsillectomy can be performed in patients with recurrent peritonsillar abscess or as an interval procedure after acute infection has resolved. Furthermore, 5–10% of DNSAs have otogenic origins, such as acute otitis media, chronic suppurative otitis media, and mastoiditis that spread beyond the tip or through its septum (Bezold abscess) into the sternocleidomastoid region and deep spaces of the neck [9,26]. Pathogens implicated include Streptococcus pneumoniae, Haemophilus influenzae, Pseudomonas aeruginosa, and anaerobes. In chronic infections, antibiotics effective against pseudomonas and mastoidectomy are needed.

Sinonasal sources are less frequent and account for 2–5% of the cases, which mostly include complicated acute or chronic sinusitis (SciELO), particularly affecting those who are at risk of extension to the orbit or intracranium [9]. Pathogens are Streptococcus pneumoniae, Haemophilus influenzae, Moraxella catarrhalis, and anaerobes. Source control might necessitate endoscopic sinus surgery. Infections in salivary glands, such as suppurative parotitis and submandibular sialadenitis, can spread into the surrounding deep spaces of the neck. Staphylococcus aureus accounts for most acute suppurative parotitis infections, and streptococci and oral anaerobes are frequent with submandibular gland infections [27]. Iatrogenic and trauma-induced sources: postprocedural infections after dental extraction, tonsillectomy, endoscopy, or swallowing of a foreign body. Penetrating neck injury can convey skin flora and pathogens of the environment. Such infections may be due to non-classical organisms such as Gram-negative bacilli requiring specific antibiotic coverage according to their mechanism and origin of contamination [8,9]. Idiopathic cases with no known primary site are described in 10–20% of series despite extensive work-up, emphasizing the diagnostic difficulty [8,18]. Such cases should be assessed with respect to occult dental pathology, immunodeficiency, or malignancy. There are geographic and healthcare setting-related differences in etiology distribution. Odontogenic etiologies are typically more common in LMIC and settings with poor access to dental care, whereas pharyngotonsillar causes are more proportionally likely in the pediatric population in high-income countries, which have established systems of dental health [7,22].

#### 3.2.2. Risk Factors and Predisposing Conditions

The pathogenesis of DNSA is associated with various environmental, medical, and genetic factors. Lower levels of education, delayed access to care, and living in rural areas are associated with more severe disease and ICU admission [10]. Chronic periodontitis, frequently attributed to poor oral hygiene and restricted dental service access, is a well-documented risk factor. Of the medical comorbidities, diabetes mellitus is the most universally linked risk factor, considering it is associated with impaired immune function, altered microbiota, and poor glycemic control, leading to further spread of infection. Immunocompromised patients (e.g., HIV-positive, chemotherapy-treated, taking corticosteroids) suffer complications and death at increased rates [17]. Cardiovascular disease, chronic renal or liver disease, and substance use (notably alcohol and smoking) also increased risk [17,18]. A role for host genetic predisposition has been hypothesized but currently lacks direct evidence in DNSAs. Future biomarker-driven studies may clarify whether genomic variability influences disease severity or response to therapy. Regional variation in allele frequency might lead to region-specific patterns of disease severity or antibiotic tolerance (e.g., a higher prevalence of slow acetylator phenotypes in South American or East Asian populations). These findings should be incorporated into the design of DNSA management programs, but additional research is required. Age continues to be a significant influencer; young children have a disproportionately high rate of retropharyngeal abscesses owing to lymphatic anatomy, and older individuals are at much higher risk due to immunosenescence and multimorbidity. Symptoms are typical in neither group and may be late occurring, leading to difficulties in diagnosis and increased rates of complications [3,6,12,29].

### 3.3. Microbiology and Pathophysiology

#### 3.3.1. Microbiological Profile

DNSAs are almost uniformly polymicrobial, reflecting a complex mixture of aerobes and anaerobes derived from the oropharyngeal and oral flora [8,9]. Common aerobic pathogens include the Streptococcus milleri group and Streptococcus pyogenes, often co-isolated with Staphylococcus aureus, including methicillin-resistant strains (MRSA) [8,9]. Gram-negative organisms—especially Klebsiella pneumoniae—emerge more frequently in diabetic or immunocompromised hosts [8,11,12]. Anaerobic bacteria, such as Peptostreptococcus, Prevotella, Fusobacterium, and Bacteroides, are also prevalent, particularly in odontogenic abscesses [8,9]. Microbiological profiles vary regionally; for instance, Gram-negative dominance is more common in Southeast Asia and LMICs, while oral streptococci predominate in HICs [9,10,29]. The emergence of antimicrobial resistance—including beta-lactamase production among anaerobes, MRSA, and multidrug-resistant Gram-negatives—complicates empiric therapy and necessitates broad-spectrum or guided antibiotic regimens [9,10,33].

#### 3.3.2. Pathophysiology and Spread

DNSA pathophysiology hinges on the unique anatomy of the neck’s fascial planes, which facilitate rapid extension of infection. Biofilm formation on infected teeth or pharyngeal surfaces contributes to chronicity and antibiotic tolerance [8]. Once an abscess breaches fascia, it propagates along potential spaces: pretracheal, lateral pharyngeal, and retropharyngeal planes—pathways that permit descending spread into the superior and posterior mediastinum, risking descending necrotizing mediastinitis (DNM) with high mortality [5,18,34]. Vascular complications—such as internal jugular vein thrombosis and carotid sheath invasion—are associated with aerobic–anaerobic synergy and biofilm bacteria [18]. Airway compromise emerges through mechanical compression, edema, and descending inflammation into the retropharyngeal and parapharyngeal spaces, often necessitating emergent airway interventions, including tracheostomy [35]. Neurovascular impairment and septic emboli may also ensue from advanced disease [2,5,6,17,18,26,36].

### 3.4. Clinical Presentation and Diagnostics

#### 3.4.1. Clinical Features

Patients with DNSAs typically present acutely, exhibiting the classic triad of fever, severe neck pain or stiffness, and odynophagia or dysphagia—often accompanied by stridor, drooling, voice changes, and trismus, reflecting airway compromise and involvement of masticatory muscles. The presentation varies by age group: in pediatric cases, drooling, agitation, and refusal to feed are common indicators in infants, while school-aged children often display torticollis or neck stiffness [13,14,32]. Adults typically report odynophagia and localized neck swelling, whereas elderly patients may present with atypical symptoms, such as mild sore throat or confusion, due to immunosenescence and comorbidities [6,31]. Space-specific signs include medial displacement of the tonsil in parapharyngeal abscesses, floor-of-mouth elevation in submandibular infections, and torticollis in parapharyngeal disease. Red flags necessitating urgent evaluation include rapid-onset dysphagia, dyspnea, trismus, neck asymmetry, and systemic signs such as tachycardia or fever—collectively termed the “airway disaster triad”.

#### 3.4.2. Diagnostic Approach

A focused physical examination remains the first clinical step, assessing for neck swelling, tenderness, lymphadenopathy, and signs of airway compromise; flexible nasal endoscopy can help evaluate supraglottic edema [3,37]. Laboratory tests often reveal elevated inflammatory markers, with blood cultures indicated in septic patients [33].

Imaging plays a critical role: contrast-enhanced computed tomography (CT) of the neck is the gold standard, providing accurate localization of abscess collections, identification of gas, and detection of complications such as mediastinal spread or vascular thrombosis [2,34]. While plain radiographs—like lateral neck films—can provide limited diagnostic clues, their sensitivity is inferior to CT [34]. Magnetic resonance imaging (MRI) offers superior soft-tissue contrast and is particularly valuable in detecting intracranial extension or skull-base involvement; however, its use is limited in unstable patients due to lengthier acquisition times [34].

Nonetheless, it is critical to realize that CT imaging has recognized limitations in differentiating drainable abscess from phlegmonous cellulitis or early suppurative lymphadenitis. Although the presence of a hypodense center with peripheral rim enhancement is a classic CT sign and suggestive of an abscess, it has been reported to have false-positive rates ranging from 10% to 25% in the literature [43]. In a retrospective analysis of 162 patients for suspected deep neck abscess, Chuang et al. showed that CT scanning had a positive predictive value of 82% for surgically proven abscess, and in as many as 18% of cases, patients went to the OR with only phlegmon or inflamed tissue without drainable pus based on CT imaging [43]. Similarly, it has been described in pediatric studies that up to 25% of children brought to the operating room based on CT findings suggestive of abscess do not have drainable collections at surgery [14,19].

CT diagnostic ambiguity is due to a number of factors. In predrainage, the rim enhancement is often present around foci of tissue necrosis, but before true liquefaction occurs; therefore, early abscesses may have a similar appearance to cellulitis. In small non-mature abscesses (30% for collections 2.5–3 cm), the presence of gas in the collection, failure to improve after 48–72 h of appropriate intravenous antibiotics, and both intra-abdominal conditions and the patient’s condition are worsening. Conversely, small collections with indeterminate rim enhancement in patients who show clinical improvement on medical therapy may be better managed conservatively with serial imaging than by surgical intervention [19,22]. Radiation can also be a concern, with potential for radiation-induced cancer, particularly in the pediatric population. One contrast-enhanced neck CT scan provides a radiation dose of 3–6 mSv, and children with DNSAs frequently receive multiple imaging studies within the course of their clinical condition [2]. The combined radiation exposure in these settings highlights the need for prudent CT use and consideration of risk-reduction strategies when appropriate.

##### Ultrasound as a Radiation—Sparing Alternative

Ultrasonography has recently become an even more valuable method for evaluation of DNSA because it has a number of potential advantages over CT; namely, there is no ionizing radiation exposure, most patients do not require sedation to complete the examination, they can be observed dynamically in real time, they can be repeated multiple times after treatment for response monitoring, and it is less expensive than CT [24,38]. These features render US particularly useful in certain clinical settings and patient populations.

##### Diagnostic Performance of Ultrasound

Led by experienced operators, ultrasound shows excellent sensitivity (70–85%) and specificity (80–90%) in the detection of superficial and lateral neck abscesses [39]. Ultrasound is effective in detecting collections, assessing internal echogenicity to differentiate an abscess from a solid lymphadenopathy, defining collection size and the presence of loculation, as well as showing associated inflammatory changes. Color Doppler study, along with peripheral vascularity, can add to the discrimination between abscess (avascular center and echogenic ring with hyperemic halo) from hypervascular lymphadenitis. However, ultrasound has well-documented limitations. Deep spaces—retropharyngeal, prevertebral, and deep parapharyngeal—are not well demonstrated because of acoustic shadowing from the mandible, hyoid bone, and air in the pharynx. Sensitivity for the detection of retropharyngeal abscess is 40–50%, so US cannot be used as a stand-alone modality in cases with deep space involvement [38]. For the same reason, ultrasound cannot accurately determine mediastinal involvement and vascular complications, such as internal jugular vein thrombosis.

##### Patient Selection for an Ultrasound—First Strategy

On the basis of current supportive data, a US-first imaging approach may be considered in the following clinical settings:

- Pediatric patients suspected of having a superficial or lateral neck abscess.

Children with presumed superficial (lateral) neck abscess, especially if the clinical examination suggests submandibular, anterior cervical, and superficial parotid space involvement, can be evaluated first by US. This strategy circumvents exposure to radiation and the need for sedation that both CT care interventions require in little children. If ultrasound shows a circumscribed and accessible collection of fluid, ultrasound-guided aspiration or drainage is possible without the need for a CT scan [14,24].

- Clinically stable patients with localized lateral neck swelling.

Patients of any age who have focal, superficial lateral neck swelling without evidence of airway obstruction, systemic toxicity, or deep space involvement can be evaluated with an initial ultrasound. A superficial abscess that is clearly defined by ultrasound can be treated with an ultrasound-guided drainage, and CT can be reserved for situations of diagnostic doubt or therapeutic failure.

- Resource-poor settings without the presence of CT.

In centers where access to CT can take place only after a prolonged time or it is not available, ultrasound appears to offer reliable diagnostic information that may be useful in deciding on initial management options, i.e., the need for empiric surgical drainage or referral [38,39].

- Serial assessment of treatment response.

Ultrasound is ideal for serial image acquisition to evaluate response to medical therapy or remaining lesions following drainage without having the patient be exposed to multiple CTs [24].

Pregnant patients.

When DNSA is suspected in pregnant women, ultrasound should be the first imaging technique used to avoid fetal exposure to radiation, with MRI performed for further investigation.

Criteria Favoring CT Over Ultrasound

On the other hand, CT remains the preferred initial imaging method in the following:

- Clinical suspicion of retropharyngeal, prevertebral, or danger space involvement;
- Signs of airway obstruction that necessitate immediate evaluation for the patency of the airway;
- Mediastinal extension or descending infection should be suspected;
- Clinical signs of vascular involvement (Lemierre syndrome, carotid);
- Trismus with mouth opening and intraoral examination restricted;
- Poor response to initial medical therapy, necessitating extensive surgery planning.

Point-of-Care Ultrasound (POCUS)

Point-of-care ultrasound, either by an emergency physician or otolaryngologist, is now being increasingly used as a quick initial assessment [38]. POCUS can rapidly recognize drainable collections on unstable patients and direct immediate bedside aspiration, aiding in management decision-making about the airway. However, POCUS is not a substitute; it should be considered as an adjunct to formal imaging in the setting of suspected deep space involvement. Teaching requirements and qualifications to use POCUS for deep neck infections have not been established, and diagnostic effectiveness is operator-dependent.

Integrated Imaging Approach

With a clinical–pathologic correlation, the imaging of DNSA should employ an evidence-based approach together with modality determination. For patients in whom ultrasound-first evaluation is appropriate and a negative or equivocal sonogram occurs with high clinical suspicion, one should not be falsely reassured and should proceed to CT imaging. Conversely, demonstration of a superficial, easily defined collection on US in the clinically stable patient may reduce radiation exposure, cost, and time to drain insertion. This individualized, sequential process maximizes diagnostic yield while minimizing unnecessary imaging and the associated risks [24,38,39,40,41,42].

Diagnostic challenges and pitfalls include early-stage cellulitis mimicking abscess on CT, deep fascia obscuring fluctuance on physical exam, and imaging misinterpretation between necrotic lymph nodes and abscesses on MRI [43]. Hence, clinical correlation is essential. A low threshold for advanced imaging should be maintained when red flags appear, given the high stakes of airway compromise and mediastinitis.

In view of these constraints, imaging findings should never be considered in isolation. Clinical course, such as response to initial antibiotic treatment, progression of symptoms, and systemic inflammatory markers, is crucial for the interpretation of imaging. In equivocal cases, a 24–48 h trial of intravenous antibiotics with careful clinical observation before deciding on surgical management is also a reasonable approach, as long as the patient does not exhibit significant disease progression, persistent fever, or an increasing collection on repeat imaging [19,22]. This approach would decrease the number of unnecessary operations and yet allow for prompt intervention in cases of true abscess. A low threshold for advanced imaging in the context of red flags should be maintained, considering implications of airway compromise and mediastinitis; however, the decision to proceed with surgery drainage ought to incorporate both imaging findings and physical exam findings rather than judging based on CT appearance exclusively.

### 3.5. Management Strategies

#### 3.5.1. Medical Management

Like most precision medicine tools, rapid molecular diagnostics and AI-based triage systems are unvalidated in DNSAs; subsections that follow have been shortened and reframed to convey only those approaches that are informed by DNSA-specific or closely related infectious disease evidence. Anything beyond this simply has to be presented, transparently, in the form of future research questions.

**Figure 2:**
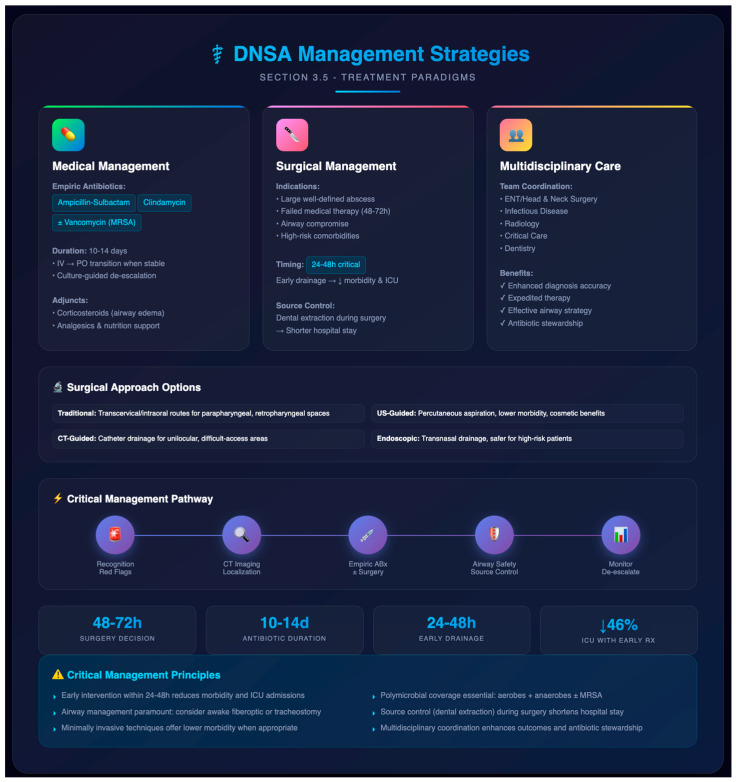
DNSA management strategies: a conceptual clinical framework. This figure presents a conceptual framework illustrating the multimodal management approach for deep neck space abscesses, organized by the authors based on narrative synthesis of treatment paradigms reported across included studies. The diagram depicts the interrelationship between medical management (empiric antibiotic selection, culture-guided de-escalation), surgical intervention (timing, approach selection), and multidisciplinary coordination. While therapeutic principles depicted are evidence-informed and reference the primary literature [6,8,9,10,17,22,38], the visual organization, decision pathways, and hierarchical structure represent author-generated schematic synthesis rather than a validated clinical algorithm or quantitative decision model.

Empiric antibiotic therapy remains the cornerstone of early management in DNSAs, with regimens targeting common oral flora (aerobic streptococci, anaerobes, and Staphylococcus aureus and MRSA where prevalent) and tailored to local resistance patterns [8,9,10]. Typical empiric regimens include ampicillin–sulbactam or clindamycin, sometimes combined with vancomycin or ceftriaxone when MRSA or Gram-negative coverage is necessary, especially in high-risk or immunocompromised patients [6,10] (Figure 2).

Once culture results are available from aspiration or intraoperative specimens, de-escalation to narrow-spectrum, pathogen-directed antibiotics is recommended to reduce collateral damage and optimize outcomes [9]. Therapy duration typically spans 10–14 days, beginning intravenously and transitioning to oral agents when clinical improvement and normalization of inflammatory markers are achieved. Adjunctive measures—such as corticosteroids to reduce airway edema, analgesics, and nutritional support—may be employed case by case, although high-level evidence remains limited [17].

#### 3.5.2. Surgical Management

Surgical intervention is indicated in patients with large, well-defined abscesses, failure to respond after 48–72 h of antibiotics, airway compromise, or high-risk comorbidities [17,22]. The timing of surgery is critical: early incision and drainage within 24–48 h has been linked to reduced morbidity and ICU admissions compared to delayed intervention [2]. Traditional surgical approaches depend on abscess location, utilizing transcervical or intraoral routes for spaces like parapharyngeal, retropharyngeal, or submandibular regions [44]. Minimally invasive techniques have gained traction—ultrasound-guided percutaneous aspiration and drainage offer lower morbidity, reduced hospital stay, and cosmetic benefits in well-circumscribed abscesses [38]. CT- or image-guided catheter drainage is effective for unilocular collections and difficult-to-access areas like the infratemporal fossa [17]. Endoscopic drainage (e.g., transnasal) is emerging as a safe alternative in selected cases, especially when external approaches pose higher risks or in medically vulnerable patients [45]. Airway management is paramount during surgical planning. In cases of threatened airway, awake fiberoptic intubation or elective tracheostomy may be necessary, particularly in Ludwig’s angina or extensive submandibular involvement [26,31]. Source control also includes addressing the primary origin, such as dental or tonsillar foci. Early removal of infected teeth—ideally during initial surgical intervention—has been associated with shorter hospital stays and reduced risk of recurrence [2,6]. Surgical decisions should be individualized based on abscess location, patient risk factors, and airway status.

##### Minimally Invasive and Image-Guided Surgical Approaches

The surgical treatment of DNSA has changed with the advancement in imaging and instruments, which led to more individualized approaches according to abscess location, size, and patients. The percutaneous aspiration and drainage under ultrasound guidance of unilocular abscess is described in several case series [38]. Benefits include decreased surgical morbidity compared to open transcervical drainage, avoidance of general anesthesia in some instances, shorter hospitalization (reported series demonstrate a mean decrease in stay of 1.8–3.2 days), and better cosmesis.

Minimally invasive drainage—including ultrasound-guided needle aspiration, ultrasound-guided catheter drainage, CT-guided drainage, and the endoscopic transoral approach—has been a useful alternative to traditional open transcervical surgery in well-selected patients [38,42].

The minimally invasive approach is based on the assumption that source control may often be obtained by drainage of purulent collections with high rates of success and a lower morbidity when compared to open surgical exploration. These techniques present several potential advantages in selected patients, which are as follows: low tissue trauma and surgical morbidity; low postoperative pain intensity; avoiding general anesthesia in some cases; faster clinical recovery time; decreased length of hospitalization; better aesthetic results, without or with minimal external scars compared to those of open surgery; patients who are at high risk due to comorbidities can undergo a procedure for primary treatment; and ability for repeat intervention if necessary [38]. Of note, several case series have described that clinical resolution (defervescence, decreased pain and swelling, normalization of white blood cell count) may be observed more rapidly after percutaneous drainage as compared to open surgical drainage techniques, presumably reflecting less perioperative trauma and inflammatory stress [38,39].

In a single-center retrospective comparative study conducted in Spain, Limardo et al. retrospectively reviewed a total of 62 adult consecutive DNSA patients from 2015 to 2020 and compared those who underwent percutaneous ultrasound (US)-guided drainage (n = 28) with surgical open drainage (n = 34) [38]. Response to treatment was defined as resolution of clinical symptoms without a need for further intervention and progressed similarly between the two groups (US-guided: 24/28 [85.7%] versus surgical: 31/34 [91.2%]; *p* = 0.67, Fisher’s exact test). Mean length of hospital stay was significantly reduced in the US-guided group (6.2 ± 2.1 vs. 8.7 ± 3.4 days; *p* = 0.04, Student’s *t*-test). Selection bias does, however, restrict interpretation since patients with multiloculated or anatomically complex abscesses were preferentially treated by surgery.

However, interpretation is limited by selection bias, as those patients with complex, multiloculated, or anatomically challenging abscesses were managed preferentially surgically. CT-guided catheter insertion has been described for abscesses in nonreadily accessible sites, such as the infratemporal fossa and deep parapharyngeal space, but only from small case series. In selected cases, transnasal endoscopic drainage of retropharyngeal and parapharyngeal abscesses has become an alternative to external approaches [42]. Potential benefits may include no facial cuts, direct visualization of the abscess cavity, and management of concomitant sinus pathology. Endoscopic drainage, however, is a technique that needs specific materials and training, but there are no comparative effectiveness data. Most of the reports mention applications in patients not suitable for general anesthesia or external access. Choice of surgical technique should be tailored to abscess characteristics (size, location, loculation, and gas), patient’s condition (comorbidities, airway management, and coagulation), instrument, experience, and previous response. Multiloculated abscesses, patients with extension into the descending mediastinum, and patients with clinical intolerance of airway compression are best treated by open surgical drainage. Image-guided or endoscopic drainage may be considered for well-circumscribed, unilocular collections > 3 cm in size, collections in the lateral neck or superficial location that permit access under ultrasound guidance, high surgical risk patients, and salvage after failed antibiotic therapy where the patient is stable.

Imaging findings should be correlated with clinical findings to decide whether surgery is indicated, bearing in mind that CT has limitations in distinguishing a draining abscess from a phlegmon. With a reported false-positive rate of 10–25%, clinical factors should adjust the threshold for operative intervention when diagnosed by CT [40]. Patients with large collections (>2.5–3 cm), air in the collection, or clinical deterioration on appropriate antibiotic therapy should undergo surgical drainage despite unclear findings on imaging. Patients with small or indeterminate collections who respond to medical therapy may be observed further and reimaged at 48–72 h.

Diagnostic and therapeutic indications of ultrasound-guided needle aspiration apply to superficial and lateral neck collections readily visualized by ultrasound. Aspiration of purulent material, which confirms the diagnosis, may be a therapeutic measure for small uninoculated collections, with a dry tap or serous aspirate indicating phlegmon and supporting systemic management [39]. This method may decrease unnecessary open surgical procedures without compromising a definitive diagnosis. In cases where percutaneous aspiration guided by imaging is non-diagnostic or results in scant fluid despite imaging data suggestive of an abscess, the clinical decision to proceed with open surgical exploration should take precedence.

There have been no randomized trials comparing minimally invasive to open surgical procedures. Published case series are at risk of bias and do not utilize standardized outcomes. Our recommendations for future research are to establish patient selection criteria for minimally invasive procedures; report outcomes with a standardized set of measurements, including success rate, recurrence, complications, and hospital stay; assess cost-effectiveness; and determine learning curve and training needs. Decision-making about the approach of incision of abscesses is based on overall clinical examination, which involves not only the features of abscess (size, site) but also patient and institutional factors, and it is subjective.

##### Ultrasound-Drainage: Procedure, Indication, and Result

Of the minimally invasive techniques, USG-guided drainage is increasingly proposed because it is easy to perform with real-time imaging visualization (which is not associated with radiation) and has proved effective in certain patients [39,40,41]. This series includes a comprehensive review of ultrasound-based techniques, as well as evidence-based patient selection criteria and comparative outcomes.

Technical Approaches

Ultrasound-guided drainage includes two main methods, needle aspiration and catheter drainage, which must be decided depending on the size of the collection and contents’ viscosity, especially to look for reaccumulation or clinical evolution [41].

US-guided needle aspiration is performed by insertion of an 18- or 16-gauge needle into the abscess cavity under real-time US guidance and aspiration of all purulent content. It is a bedside emergency and outpatient procedure that can be carried out under local anesthesia [40]. Aspirated material should be submitted for Gram stain and culture to direct antibiotic therapy, as new data support the critical role of microbiological identification in successful therapy [42]. Smaller collections (5 cm) are more likely to require open drainage for proper source control [41]. A non-vascular and anatomically less critical sonographic window should be present in the needle path, and a lack of gas in the abscess is preferable as gas can indicate an anaerobic infection, which has been associated with high failure rates [42].

Clinically, the following are considered favorable: a stable patient without sepsis or hemodynamic compromise, an airway that is not compromised (i.e., no stridor and severe dyspnea or rapidly progressive swelling), and no trismus or trismus that allows safe positioning [40]. There must be no clinical or radiological suspicion of deep space extension, such as retropharyngeal, danger space, or prevertebral involvement, and evidence of complications referring to mediastinal extension, thrombophlebitis, or necrotizing fasciitis [40,41,42]. The patient must be able to feel and follow procedural instructions or be a candidate for procedural sedation.

Favorable patient variables that lend towards US-guided drainage include high surgical risk based on comorbidities such as severe cardiac or pulmonary disease, coagulopathy, case specific consideration (patient’s preference to avoid open surgery in clinically suitable cases), pediatric clinical scenario where avoidance of general anesthesia is desired and pregnant clinical settings where reduction of surgical intervention is more acceptable and preferable [39,41].

Contraindications to Ultrasound-Guided Drainage

It should be noted that in various scenarios, US-guided drainage is not suitable, with classical open surgical drainage being the treatment of choice [40,41]. These are (1) airway compromise requiring emergency surgical airway management; (2) deep space involvement not amenable to percutaneous approaches, including retropharyngeal, prevertebral, or danger space infection; and (3) facial necrotizing fasciitis suspected or proven descending necrotizing mediastinitis [40]. Other contraindications are multiloculated collections with septations that were thick and considered unlikely to be drained percutaneously, clinical suspicion of necrotizing fasciitis for which surgical debridement was needed, and locations near major vessels such as the carotid artery or internal jugular vein where there was no safe needle trajectory [41]. Previous unsuccessful US-guided drainage with worsening clinical condition and presence of foreign body or underlying pathology, such as branchial cleft cysts or necrotic tumor in need of definitive curative surgery, also contraindicate minimally invasive procedures [40].

Comparative Results: US-Guided versus Open Surgical Drainage

A few retrospective studies showed similar results of the US-guided and the open procedure, but no RCT was available [39,41]. Available evidence indicates similar effectiveness with potential benefits for minimally invasive treatments in suitable patients. Limardo et al., in a Spanish single-center retrospective study comparing ultrasound-guided drainage versus open surgical drainage, respectively, among 28 patients and 34 adult DNSA patients, found similar success rates of treatment (85.7% in comparison to 91.2%, *p* = 0.67). Nevertheless, hospitalization was significantly shorter for the ultrasound-guided group—6.2 ± 2.1 days as opposed to 8.7 ± 3.4 days (*p* = 0.04). Time to clinical improvement (defervescence plus >50% decrease in neck mass) was also shorter with ultrasonography guidance than traditional incision and drainage (mean time 2.1 versus 3.4 days; *p* = 0.03), indicating that minimally invasive drainage accomplishes faster source control with less procedure-related inflammation.

Fan and Tao reported a comparative analysis comparing US-from incision-guided puncture drainage to deep neck abscess based on the evidence suggesting that ultrasound-guided methods had similar clinical success rates with less associated functional injury and faster recovery when applied in the proper population [41]. Their results are in line with the increasing evidence that minimally invasive approaches should be used as primary procedures for uncomplicated cases.

With respect to clinical success rates, the published series for ultrasound-guided aspiration or catheter drainage report success between 75% and 92%, defined as resolution without requirement for subsequent open surgery [39,41]. Lower success is reported in collections greater than 4 cm, multi-loculated abscesses, and immunosuppressed patients [39,42]. Febrile response following US-guided drainage is defervescent in 24–48 h in the vast majority of patients, as opposed to 48–72 h after open surgical drainage, and this early clinical response may be a measure that it leads to less tissue trauma and generalized systemic inflammatory reaction [39,41]. A decrease in the average hospital stay of 1.8–3.2 days has been reported with ultrasound-guided (USG) vs. open drainage in a number of series, resulting in significant cost savings and lower exposure to hospital-acquired complications [39,41]. In the neck region, 15–25% of patients require re-aspiration or conversion to open surgery after ultrasound-guided aspiration, due to re-accumulation or incomplete drainage, while catheter drainages are associated with lower reintervention rates (8–15%) in comparison to needle aspiration only [39,41]. Ultrasound-guided drainage has a reported rate of complications at <5%, which were primarily comprising minor bleeding and transient increased pain, with an occasional procedure-related infection spread, other major complications such as vascular trauma or nerve injury occurring rarely when patients are selected appropriately and when the procedures are experienced [41].

Practical Implementation Considerations

The success of US-guided DNSA drainage depends on the institution’s interest in training, resources, and multidisciplinary approach [40,41].

In terms of practitioner skill, ultrasound-guided drainage should be carried out by physicians trained in interventional sono/graphic techniques, including interventional radiologists, senior otolaryngologists, and emergency room doctors with experience in procedural ultrasound, and learning curves have been proposed, with 15 to 20 cases under supervision before being allowed to practice independently [39,40]. Equipment needed includes a high-frequency linear probe that ranges from 7 to 15 MHz and a low-frequency curvilinear probe for deep structures, including sterile probe coverings, needles (18 or 16 gauge), drainage catheters, triangular-tipped of cross-sectional diameter of 8–12 French pigtail configuration, and local anesthetic [41]. Procedures may be conducted at bedside, in the emergency department (ED), interventional radiology suite, or operating room according to patient stability and institutional preference, with the presence of emergency airway equipment and staff being crucial [40]. Patients undergoing drainage by ultrasound guidance need close clinical follow-up with a repeated ultrasound at 24–48 h to find out whether there is any remaining or reaccumulated collection, and lack of improvement within 48 h must prompt further evaluation and consideration of open surgery for drainage [39,41].

Pediatric Considerations

Ultrasound drainage, in particular, is an attractive option for pediatric patients as it avoids radiation exposure and may avoid the need for general anesthesia [40]. If cooperative, older children and adolescents may be able to undergo needle aspiration under local anesthesia with or without anxiolysis; however, younger patients would need procedural sedation, although this approach still prevents the risks associated with general anesthesia and endotracheal intubation. Ultrasound-guided aspiration has been successful in 70–85% of children with superficial neck abscesses in previous pediatric case series, although some needed open drainage [24]. The indication for PETS in children should be performed very selectively and with a low-threshold conversion to open surgery in the absence of prompt clinical amelioration [40].

Integration into Clinical Practice

According to the current evidence, we suggest a step-by-step protocol of US-guided drainage in the treatment of DNSA [39,40,41]. There is also a potential role for all patients with presumed DNSA to be clinically assessed for airway stability, sepsis criteria, and extent of anatomic infection [40,42]. Those with airway compromise, septic shock, suspected deep space or mediastinal involvement, or necrotizing infection should go straight to open surgical drainage with possible airway management [40]. For clinically stable patients with a superficial or lateral neck abscess that fulfils the selection criteria (unilocular collection in an accessible location of 20–5 cm without any complexity), ultrasound-guided drainage should be weighed as appropriate for first-line intervention [41]. US-guided needle aspiration may be applied in a small collection of 2–3 cm, as well as the catheter drainage for large collections, such as 3–5 cm or viscous appearances. Near clinical and sonographic monitoring is essential at 24–48 h, without hesitating to repeat intervention with no delay or open conversion for insufficiency of effect [39,41]. All should receive concurrent IV antibiotic therapy irrespective of the drainage mode and de-escalation on culture reports based on the microbiological characterization [42].

This phased individualized approach has the potential to minimize surgical morbidity and hasten clinical recovery of these patients with a corona-like COVID-19 disease and promote the rapid emergence from pandemic responses, with continued access to timely definitive open surgical intervention for those who need it [39,40,41].

#### 3.5.3. Multidisciplinary Care Coordination

Optimal DNSA management demands coordinated multidisciplinary input, often involving otolaryngology/head and neck surgery, infectious diseases, radiology, critical care, and dentistry. This collaborative structure enhances diagnosis accuracy, expedites definitive therapy, ensures effective airway strategies, and supports tailored antibiotic stewardship [1,6,46].

### 3.6. Outcomes and Global Burden

#### 3.6.1. Clinical Outcomes

DNSAs frequently necessitate prolonged hospitalization, with mean stays ranging from one to more than two weeks, depending on patient age, abscess size, and treatment modality. A national USA pediatric study reported an average length of stay (LOS) of 3.4 days for medical management and 4.2 days for surgical drainage, contributing to total hospital charges over USD 75 million in 2009 [13,21]. Adult cohorts with mediastinal involvement often experience a substantially longer LOS—averaging nearly 40 days, compared to approximately 10 days in non-mediator patients [5]. ICU utilization is notable in severe cases, with studies showing 46% of patients requiring ICU-level care often due to airway compromise or systemic sepsis [5]. Readmission rates are underreported but may be elevated in those with incomplete source control or comorbidities. Functional outcomes are rarely studied longitudinally; however, surviving patients often face prolonged recovery, persistent neck stiffness, dysphagia, or diminished quality of life, particularly in those with complicated thoracic extension or vascular sequelae [31,32].

#### 3.6.2. Mortality and Morbidity

DNSA mortality rates differ according to setting and case difficulty. In the Finnish population cohort study (n = 277 patients), overall mortality was 1.6% (4/277 patients) [6]. The UK tertiary center retrospective series (n = 53) had 7.5% mortality (4/53), and only patients with mediastinal extension died [32]. Mortality rises to high levels in descending necrotizing mediastinitis. A German retrospective series of 218 deep neck infections reported a mortality rate of 40% (8/20) in the subset that had mediastinal involvement versus 1% (2/198) that did not [3].

Severe life-threatening complications include obstruction of the airway, descending necrotizing mediastinitis (DNM), and septic thrombophlebitis (including Lemierre’s syndrome) or neurologic sequelae. Prognostic factors for unfavorable outcomes have been discovered in different populations: diabetes mellitus, multi-space disease, and odontogenic etiology were independent risk factors for ICU admission in the Finnish cohort (OR = 2.0–3.4) [6]. In a retrospective cohort of 89 adult DNSA patients from Mexico, García-López et al. found age > 60 years (OR 3.2; 95% CI 1.4–7.3), diabetes (OR 4.1; 95% CI:1.8–9.4), and ≥2 anatomical spaces involved (OR = 5.7; 95% CI 2.3–14.1) to be independently associated with complications by multivariable logistic regression [11].

#### 3.6.3. Economic Impact

**Figure 3:**
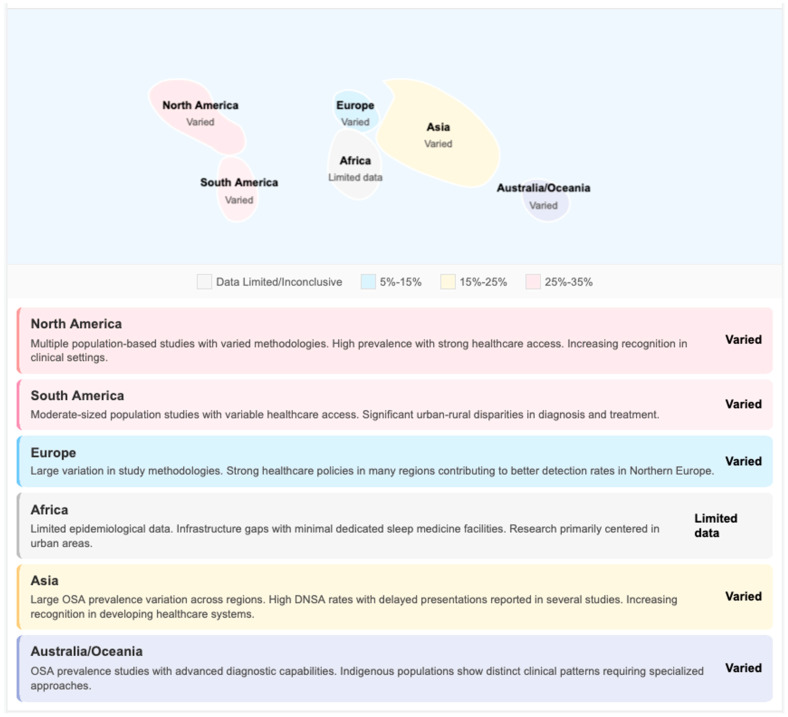
DNSA economic impact and regional variations: a hybrid data summary. This figure combines quantitative economic data extracted from included studies with a schematic geographic framework to illustrate regional variations in DNSA-associated healthcare costs and resource utilization. Numerical data presented (e.g., hospitalization costs, length of stay, ICU utilization rates) are derived directly from cited primary studies and represent reported values rather than author-calculated estimates.

DNSAs impose a significant economic burden. In the United States, pediatric cases alone accounted for over USD 75 million in total hospital charges in 2009, with surgically treated patients incurring nearly twice the costs of those managed medically [21]. Indirect costs—including lost productivity, caregiver burden, and long-term morbidity—are less quantifiable but likely substantial (Figure 3).

Although we have included data from multiple countries in North America and Europe, there is a paucity of population—level data on the burden of DNSA in Central and South America, including Mexico and the Caribbean regions. For instance, a Brazilian series of 101 cases of deep neck abscesses offers a clinical guideline but does not apply for epidemiologic generalization to the South American region. Hence, some places like Mexico, Guatemala, Colombia, Brazil, or Argentina now appear undersampled in the comparative HIC/LMIC framework. Hence, we emphasize the importance of future multicenter epidemiology in Latin America to fill this knowledge gap and facilitate a better comparison of DNSA burden, outcomes, and resource consumption at the global level. While few formal cost-effectiveness analyses exist, studies increasingly advocate primary dental care access and early drainage strategies as cost-saving measures, particularly when offsetting ICU utilization and extended hospitalization [16,30].

#### 3.6.4. A Precision Public Health Perspective

To alleviate the burden of DNSA, interventions at several tiers of the system, from individual clinical care to population-level prevention, are needed. Some health systems have introduced clinical pathways to help identify DNSA early and route patients appropriately. ED red-flag symptom checklists for fever >38.5 °C, progressive dysphagia, neck swelling, and trismus have been shown in pilot studies to be associated with decreased time to CT imaging and otolaryngology review [3]. Education strategies in primary care targeted at early recognition and referral appear to be promising but are poorly evaluated. Networks of telemedicine consultations from rural physicians to tertiary ENT providers have been set up in Australia and Canada, facilitating better access to specialist input for diagnostic and management solutions [30]. With the high ratio of odontogenic origins, dental public health programs are pivotal in preventing CSF/RL subsites. Community water fluoridation and school-based fluoride programs have been successfully demonstrated in the prevention of dental caries, the main precursor of odontogenic DNSA [16]. Increased availability of preventive dental services in underserved populations, such as rural, low-income, or Indigenous communities, is associated with lower rates of severe odontogenic infections; however, specific DNSA impact data are sparse. Immunization programs against pneumococci and Haemophilus influenzae type B have led to a decrease in the number of pharyngotonsillitis infections; as an indirect consequence, this caused a reduction also of DNSA pediatric cases [46,47,48]. At least in settings with resource constraints, some targeted investments could bring high returns. This involves the need for contrast-enhanced CT, and for regional hospitals, this is not readily available (for more than 70% of East African regional facilities according to WHO surveys [34]), which is a prerequisite. There is a critical need to develop training programs for general surgeons in DNSA drainage techniques in settings where ENT surgeons are not readily available. Hospitals managing DNSA should develop ICU beds and establish airway management protocols, which will reduce avoidable mortality. Antimicrobial stewardship efforts targeting empiric regimen optimization and monitoring of resistance are important. The lack of routine DNSA surveillance hinders the appreciation of actual disease burden, temporal changes, and intervention success. Priority actions include the establishment of standardized DNSA case definitions for public health surveillance, pilot investigations to test hospital-based DNSA registries in representative geo-demographic sites, inclusion of data on DNSAs in existing antimicrobial resistance surveillance networks, and international cooperation to facilitate cross-national comparisons [49,50]. The highest-yield public health recommendations from the available evidence for reducing the burden of DNSA are increased access to preventive dental care specifically in high-risk populations, preservation of strong pediatric vaccination programs, development of early detection and referral processes within both primary care and emergency settings, greater healthcare system capacity-building in LMICs to enable diagnostic imaging and surgical repair, implementation of antimicrobial stewardship programs, and integration with standardized surveillance.

### 3.7. Challenges and Health Disparities

#### 3.7.1. Healthcare System Challenges

In LMICs, management of DNSAs is particularly hampered by structural system deficits. Diagnostic delays are widespread; for instance, Shouche et al. reported that over 60% of DNSA patients in a rural South African district presented more than 48 h after symptoms began, correlating with doubled rates of airway compromise and ICU transfer compared to early presenters [33]. Advanced imaging—essential for precise localization and management planning—is frequently unavailable: a World Health Organization (WHO) survey found that fewer than 30% of regional hospitals in East Africa had reliable access to contrast-enhanced CT or MRI [34]. Clinicians consequently rely on ultrasound and lateral neck radiographs, modalities associated with false-negative results in up to one-third of cases [35]. A UK tertiary center study documented significant resource utilization in DNSA management, with 32.1% requiring ICU admission and complications including mediastinitis (13.2%) and Lemierre’s syndrome (7.5%), underscoring the importance of specialized multidisciplinary care. However, such intensive and multidisciplinary management is often difficult to achieve in low- and middle-income countries (LMICs), where health systems face substantial constraints. These include limited access to intensive care facilities, delays in surgical intervention, and a critical shortage of specialized head and neck surgeons, with some regions reporting fewer than 0.5 per 100,000 population compared to more than 2 per 100,000 in high-income countries. These disparities underscore systemic challenges that can lead to delayed diagnosis, inadequate treatment, and increased morbidity and mortality associated with deep neck infections. This scarcity leads to delayed surgical drainage, increased in-hospital mortality of 4–12%, and higher complication rates such as mediastinitis and vascular thrombosis [37]. Critical care resources are similarly limited, with some hospitals lacking ventilators or ICU beds—meaning that airway compromise during transfer can prove fatal [39]. These compounded deficiencies across the patient care continuum—from symptom onset to definitive treatment—underscore the pressing need for system-wide investments in imaging accessibility, surgical training, and critical care capacity.

#### 3.7.2. Emerging Challenges

Antimicrobial resistance (AMR) is an increasing concern, which makes the challenge regarding antibiotic treatment in DNSAs more complicated. Asairinachan et al., in an Australian series of 127 DNSA patients (2015–2023), reported the cumulative growth of resistant organisms over their study period, with MRSA isolated in 12% (15/127) culture-positive cases and ESBL-producing Gram-negatives in 8% (10/127) [10]. Although direct temporal comparisons within DNSA populations are limited, regional-wide surveillance data have suggested a 35% rise in ESBL Enterobacteriaceae isolates from deep-seated infections over the last decade. Likewise, Bandol et al. found 38% (34/89) penicillin protein-resistant Staphylococcus aureus in a Romanian retrospective series of 89 deep neck infection patients (2018–2023) [8]. These trends necessitate the empirical use of carbapenems or vancomycin, which are costly and often unavailable in LMIC formularies [10,43]. The inherently polymicrobial nature of DNSAs—co-infection with both aerobic and anaerobic organisms—adds complexity, associated with longer hospital stays and increased rates of ICU and surgical interventions [6,17].

**Figure 4:**
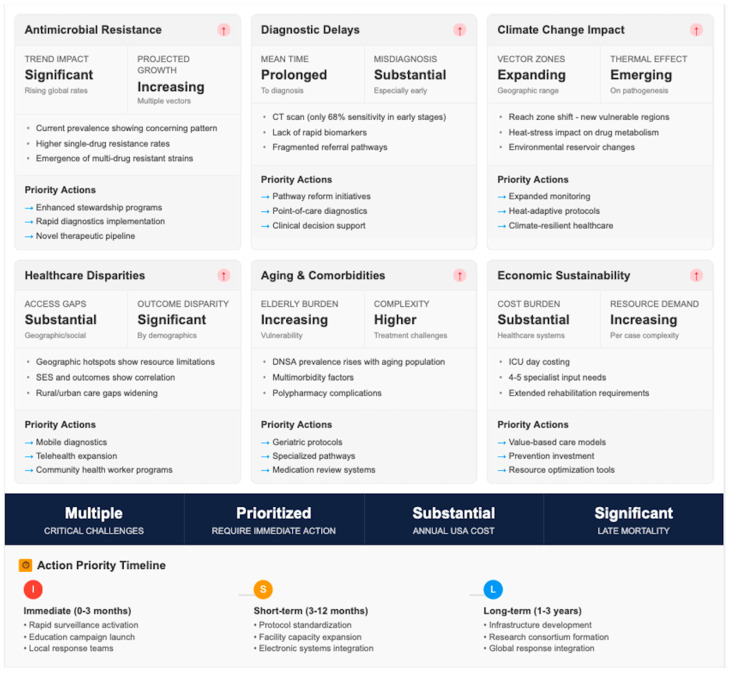
Emerging challenges and action priority timeline: an author-generated conceptual framework.

Environmental changes driven by climate change, such as increased seasonal flooding, contribute to this challenge by altering pathogen ecologies and increasing respiratory infections, which serve as precursors for DNSAs [19]. Coupled with gaps in antimicrobial stewardship infrastructure, unchecked AMR proliferation threatens to undermine decades of therapeutic progress (Figure 4).

#### 3.7.3. Vulnerable Populations

Certain demographic groups remain disproportionately impacted by DNSAs due to systemic and socioeconomic vulnerabilities.

Pediatric patients, especially infants and toddlers, are at higher risk. Adil et al. in a US national database assessed 3403 pediatric DNSA hospitalizations (2000–2009). The authors demonstrated that in comparison to older children, those less than 4 years of age had higher rates of surgical intervention (68% versus 52%, *p* < 0.001) [13]. Novis et al., analyzing the same database, reported that retropharyngeal abscesses in children under 5 years were associated with 2.5-fold higher odds of ICU admission compared to older pediatric patients (OR 2.5; 95% CI 1.8–3.5) [14].

Indigenous and minority communities demonstrate disproportionate DNSA burden.

Jamieson et al. reported that the rates of severe dental infection among Aboriginal Australians were 2–3 times that of non-Aboriginal Australians (combined rate ratio was 2.4; 95% CI = 1.9 to 3.0), which were attributed to poor access to dental care facilities, experiencing disadvantage because of socioeconomic status, and barriers within the healthcare systems [30].

Similarly, refugees and displaced populations living in crowded and unsanitary settings—such as Syrian and Rohingya refugee camps—have reported localized outbreak clusters of DNSAs with high complication rates [45,46]. Lastly, immunocompromised hosts—including people living with HIV, transplant recipients, and patients on systemic steroids—are at higher risk of polymicrobial, resistant infections and prolonged hospitalizations. European case series report a longer median hospital stay for this population, with increased ICU admission and a twofold mortality rate compared to immunocompetent patients [11].

### 3.8. Prevention and Public Health Strategies

#### 3.8.1. Primary Prevention

Effective prevention of DNSAs begins with comprehensive oral health programs. Evidence shows that individuals with regular fluoride treatment, sealant programs, and community brushing initiatives have substantially fewer odontogenic infections—a leading precursor to DNSAs—compared to communities lacking such resources [7,26]. A 2025 Finnish population study further demonstrated that individuals with poor oral hygiene were nearly twice as likely to require hospitalization for severe orofacial infections [6]. Additionally, initiatives such as school-based fluoride mouth-rinse programs have been linked to a reduction in dental caries by age 12, significantly reducing long-term DNSA risk [16].

Vaccination strategies can play a complementary role. While DNSAs are often polymicrobial, upper respiratory pathogens such as Haemophilus influenzae type B, pneumococcus, and influenza contribute to tonsillopharyngitis and lymphadenitis, which can progress to deep neck infections. Studies have shown that widespread Haemophilus influenzae type B and pneumococcal conjugate vaccination campaigns reduce pharyngotonsillar infection rates by 30–40%, indirectly lowering DNSA incidence [46].

**Figure 5:**
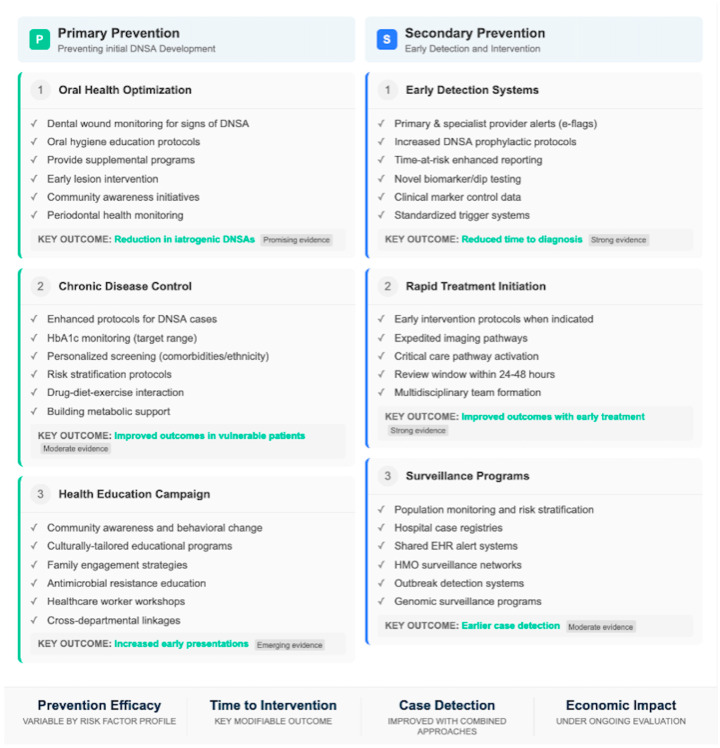
Prevention strategies for DNSA: primary and secondary measures. This table organizes prevention strategies for deep neck space abscesses into primary and secondary prevention categories. Individual strategies listed are derived from evidence reported in included studies and the broader public health literature; however, the organizational framework and categorization represent an author-generated synthesis.

Finally, improving child nutrition and immunization further enhances primary prevention. Adequate childhood nutrition—especially micronutrients like vitamin A and D—supports mucosal immunity, decreasing the severity of oral and oropharyngeal infections [16]. Full infant immunization schedules, coupling oral polio, measles, and influenza vaccines with earlier pediatric respiratory syncytial virus immunization, help prevent virus-associated mucosal damage that predisposes children to secondary bacterial invasion [47]. These combined efforts set the foundation for preventing many DNSA precursors before they begin (Figure 5).

#### 3.8.2. Secondary Prevention

To detect deep neck infections early, robust early detection protocols are essential. Standardized clinical assessment tools developed for primary care now include red-flag symptom checklists—incorporating fever, progressive neck swelling, odynophagia, and trismus—leading to significant reductions in advanced DNSA presentations by prompting urgent imaging referrals [3]. In children, criteria such as persistent fever >38.5 °C with unilateral neck swelling are highly predictive for DNSA, enabling proactive outpatient management or rapid transfer [13,14]. Community education initiatives play an integral role in bridging knowledge gaps. Targeted community education campaigns have shown promise in improving early presentation. Maharaj et al., in a South African before-and-after study comparing DNSA presentations at a tertiary hospital before (n = 48) and after (n = 52) implementation of a community awareness program, reported a reduction in delayed presentations (>48 h from symptom onset) from 62.5% (30/48) to 34.6% (18/52; *p* = 0.006, chi-square test) [22]. Similar programs in Australia and Canada have reported comparable improvements, though controlled comparative data are limited. Finally, healthcare provider training—including tele-mentorship for rural clinicians—has improved early recognition and appropriate referral. Regional quality improvement programs involving virtual case discussions and e-learning modules have reduced misdiagnosis of deep neck cellulitis in primary care by 45%, as demonstrated in LMIC pilot programs [22,46]. Multidisciplinary drills incorporating emergency physicians, dentists, and ear–nose–throat (ENT) surgeons have strengthened referral networks, ultimately improved time to drainage, and decreased complication rates [46,47,48,49].

### 3.9. Future Perspectives: Emerging Technologies and Precision Approaches

Contemporary management of DNSA is based on longstanding principles of clinical evaluation; contrast-enhanced imaging; empiric antibiotics targeted to local bacterial resistance patterns; and, when appropriate, prompt surgical drainage. The proposed strategies in this section are investigational and not uniquely validated for DNSA; these are intended as hypothetical priorities of research lines rather than suggestions.

#### 3.9.1. Rapid Molecular Diagnostics (Unvalidated in DNSA)

Other deep-seated infections can be diagnosed with pathogen detection methods like multiplex PCR panels and metagenomic next-generation sequencing within 6 to 24 h rather than 48 to 72 h for conventional culture. Detectable use cases for DNSA include early pathogen ID for guiding antibiotic de-escalation, detection of resistance genes (e.g., mecA from MRSA, bla genes for beta-lactamase), and fastidious/anaerobes that would not be detected by standard culture. Rapid molecular diagnostic modalities have not been specifically studied in DNSA populations, the cost-effectiveness and clinical value of which are yet to be evaluated prospectively but offer potential for this population.

#### 3.9.2. Biomarker-Based Risk Stratification (Hypothesis-Generating)

Recognizing patients at greatest risk for adverse outcomes such as airway obstruction, mediastinitis, or prolonged hospitalization might facilitate earlier and more aggressive treatment. Some candidate biomarkers that have been evaluated for other severe infections include procalcitonin, C-reactive protein kinetics, and so-called host-response gene expression signatures. Whether these new biomarkers specific to DNSA variants can stratify risk at presentation has not been established and deserves prospective dedicated studies.

#### 3.9.3. Pharmacogenomic Considerations (Theoretical)

Inter-individual variation in the metabolism and toxicity risk of antibiotics may be affected by population-level genetic diversity. Cytochrome P450 polymorphism (CYP2C19, CYP3A4) can influence the metabolism of macrolides and azoles, whereas (NAT2) slow acetylator phenotypes—common in some populations—can heighten risk for sulfonamide toxicity. There is potential for geographic variation in the distribution of these polymorphisms, but clinical impact has not been studied at all in DNSA. These speculations are held in anticipation of DNSA-specific pharmacogenomic investigation.

#### 3.9.4. Artificial Intelligence and Predictive Modelling (Early Development)

Machine learning models have been built to predict airway compromise and mediastinitis risk in deep space infections more broadly, but no model has been independently validated for DNSA [49,50]. AI-based clinical decision support tools that combine clinical, laboratory, and imaging data have been promising for other infectious diseases but have not yet been utilized for the management of DNSA. Efforts are also underway to develop validated DNSA-specific prediction models, which is also an active, though early, research area.

#### 3.9.5. Precision Public Health Approaches (Conceptual)

From a population perspective, precision public health interventions might theoretically maximize DNSA prevention and already limited resources. These initiatives might include, for example, targeting dental health prevention programs to high-incidence geographic regions, population-specific screening procedures, and monitoring disease in real-time to detect outbreaks. These approaches depend on strong epidemiological infrastructure and DNSA registries, which are largely absent in most areas.

#### 3.9.6. Research Priorities

Toward more personalized strategies in the care of DNSA, we propose the following research priorities:

(i) Development of multicenter DNSA registries with standardized definitions for morbidity so that the predictive model can be formulated;

(ii) Prospective assessment of rapid molecular diagnostics with abscess specimens: turnaround time, clinical utility, and applicability to the cost-of-illness model;

(iii) Validation of candidate markers to stratify severity at presentation;

(iv) The construction and external validation of clinical prediction rules for complications;

(v) Research to assess the feasibility of the use of promising new technologies in a variety of healthcare settings—particularly LMICs.

Until DNSA-specific evidence is produced from this hard work, we should not stray from the principles of prompt clinical evaluation, appropriate imaging, broad-spectrum empiric antibiotics with culture-guided de-escalation, and surgical intervention if warranted.

#### 3.9.7. Limitations in LMIC Data and Generalizability Issues

There was limited evidence from low- and middle-income (LMI) countries, which can be considered a main weakness of this review. While we sought to provide a global burden viewpoint, the evidence base from LMICs contrasts substantially with the high-income setting that is known, which limits valid inferences.

##### Evidence Asymmetry by Income Classification

**Figure 6:**
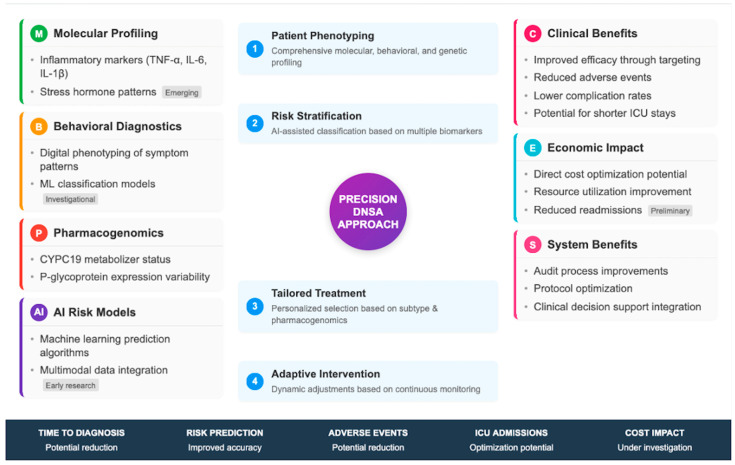
Novel technologies and precision interventions in DNSA management: a conceptual research framework. This figure presents an entirely conceptual framework illustrating potential future research directions for precision approaches in DNSA management. The diagram is author-generated and does not represent current evidence-based practice or validated clinical pathways.

Information from HICs is often provided by national registries, administrative databases, or multi-institutional collaboratives and may include thousands of cases. For instance, pediatric DNSA trends in the US were described with data from the Kids’ Inpatient Database (n = 3403 hospitalizations nationally) [21], and national Finnish population-level surveillance was available [6]. These sources allow for the estimation of true incidence, temporal trends, and population-attributable outcomes (Figure 6).

In contrast, LMIC data in this review have overwhelmingly come from single-center retrospective case series, often from the largest referral hospitals located in urban settings. Among the 16 LMIC studies meeting the inclusion criteria, 14 (87.5%) reflected single-institution experiences, and none had population-based denominators. Few studies achieved high sample numbers; the number of patients included in studies varied from 23 to 342, with most studies providing fewer than 150 cases over long timeframes (often of 5–15 years).

##### Implications for Interpretation

Single-center LMIC case series are affected by several biases and should not be generalized at the population level. Tertiary referral facilities deliberately exaggerated their complicated populations, many of them naysayers, and with resources to choose from as well. Patients were successfully managed at primary/secondary stages, and those who die before reaching referral are lost to follow-up, as well as patients with an inability to access healthcare. As a result, case series from LMIC tertiary centers probably overestimate complication rates, need for surgical intervention, and mortality as compared to actual population-level estimates.

Moreover, institutional case series mirror local customs, referral systems, antimicrobial drugs present, and surgical proficiency that might not be applicable even in similar institutions of the same nation. So, a case series from an upper-tier private hospital of the urban Indian center cannot be generalized to rural district hospitals or to other South Asian countries with different healthcare setups.

##### Specific Data Limitations by Region

Institutional case series from Sub-Saharan Africa are limited to three reports (South Africa, Nigeria, and Ethiopia) in a good number of fewer than 400 patients. No population-based African data exist. Conclusions about the DNSA epidemiology, etiology, or impact in Africa should be regarded as preliminary and hypothesis-generating rather than conclusive.

The South Asian data are mainly Indian tertiary center-based, with little contribution from Pakistan, Bangladesh, Sri Lanka, and Nepal. The considerable heterogeneity in clinical infrastructure, disease prevalence, and population demographics across this region does not allow for generalizing from the data of hospitals in India to the broader South Asian population of almost two billion people.

Evidence specifically from Latin America is limited; only two Brazilian series in the institutional setting fulfilled eligibility criteria. No published study of a case series utilizing our methodology has been reported from Central America, the Andean region, or the Caribbean.

Southeast Asian data are based on data from single centers in Malaysia, Thailand, and the Philippines, with the absence of population denominators.

##### Transparency in the Current Review

Throughout this paper, we have strived to make the source and type of evidence apparent when reporting LMIC findings. Readers need to view LMIC data as a feature of the experience at select referral centers rather than in terms of population-based epidemiology. Where we talk about LMIC results, we have hedged in such a way as to acknowledge those limitations specifically. Any disparities observed between HIC and LMIC settings that are not simply methodological artifacts or a function of differential access to and referral from healthcare could be indicative of true variation in epidemiology among subsets of the population.

We highlight that the dearth of LMIC population-based data is a significant gap in knowledge. So, absence of evidence is not automatically evidence of no effect: DNSA burden in LMICs may be far greater than described in existing institutional series. Closing this evidence gap will necessitate investment in population-based surveillance systems, multicenter collaborative networks, and standard data collection in resource-limited regions.

## 4. Discussion

In this narrative review, we provided a comprehensive overview of the global burden, etiology, microbiology, clinical presentation, diagnosis, and management of deep neck space abscesses based on an analysis of 48 studies. From these, several themes were derived.

Higher trends of DNSA rates are seen in HICs and LMICs over the 2 decades. This revival of infection may be due to a combination of factors, such as an aging population, more people with diabetes and immunosuppressive states, and possibly shifting microbial virulence profiles. The regional variation in disease burden highlights the role of access to care, social determinants, and regional disparities in oral health infrastructure.

Geographical inequalities identified by this review highlight the importance of managing root causes context-specifically. Regional microbiological trends must be considered when initiating empiric antibiotic therapy, including higher rates of MRSA in North America, dominance of Klebsiella pneumoniae in South Asian diabetic populations, and increasing resistance in ESBL-producer organisms worldwide. Strengthening of health system infrastructure in LMICs with a focus on investment for dental care, diabetes care, and surgical capacity is a public health priority to reduce burden and mortality due to DNSA in these settings [51].

Despite the majority being polymicrobial, there are worrisome trends in antimicrobial resistance with increased prevalence of MRSA and ESBL-producing organisms, thus warranting continued surveillance and selective empiric antibiotic choice based on local resistance patterns. Although contrast-enhanced CT is the gold standard for diagnosis, knowledge of its imperfections in differentiating a drainable abscess from phlegmon and radiation exposure in pediatric patient groups suggests that sonography should be used selectively as the primary imaging modality in appropriate clinical situations [52]. There is an ongoing paradigm management shift, and these minimally invasive drainage approaches are effective as open surgical treatment, provided patients are chosen carefully. The lack of randomized controlled trials results in an inability to make formal recommendations, and management plans should be considered on a case-by-case basis. Substantial differences remain in diagnostic capabilities, surgical skills, and availability of intensive care between high-income areas and low-resource settings. Targeting such deficiencies with investments in infrastructure, training programs, and telemedicine networks is a public health imperative [53].

There were several limitations with this review. Most studies were retrospective, single-center studies, which limit generalizability—especially from LMICs. Formal meta-analysis was not possible because of the heterogeneity in case definitions, measures of outcome, and duration of follow-up. The restriction to English for the language might have filtered out cogent regional information. Despite this restriction, the synthesis presents the most complete description of DNSA epidemiology and control in a variety of global environments to date.

## 5. Conclusions

DNSAs persist as a critical clinical challenge worldwide, demonstrating a clear upward trend in incidence across both high-income and low-resource settings. Population-based studies from Finland and the UK have observed a rise in case numbers over the past two decades, with annual presentations increasing from approximately 14 to 24 and 1 to 15 cases, respectively [6,13]. Similarly, retrospective data from tertiary centers in developing nations highlight not only higher baseline incidence but also more frequent delays in presentation, translating into increased complications and mortality [2,26,43].

The microbiological landscape is predominantly polymicrobial, involving Streptococcus and Staphylococcus species alongside anaerobic and Gram-negative organisms, often complicated by emerging antimicrobial resistance. Prognostically, diabetes, immunosuppression, elderly age, and multi-space involvement consistently emerge as key predictors of poorer outcomes, including a higher risk of mediastinitis, ICU admission, and increased mortality. These findings reinforce the urgent need for timely diagnosis—ideally, prompt contrast-enhanced imaging—and early multidisciplinary intervention combining medical and surgical efforts.

Moreover, these precision medicine approaches require substantial technological, financial, and human resources that are unavailable in most global healthcare settings, limiting their relevance to current worldwide DNSA management. Until DNSA-specific evidence from prospective validation studies supports these approaches, management should remain firmly grounded in established principles of early clinical recognition, prompt contrast-enhanced imaging, multidisciplinary coordination, broad-spectrum empiric antibiotics with culture-guided de-escalation, and timely surgical intervention when indicated.

Public health and systems-level enhancements are essential. Priority actions include expanding imaging and surgical capacity in underserved regions, implementing evidence-based clinical pathways, and reinforcing antimicrobial stewardship practices that address the complexities of polymicrobial and resistant infections. For example, structured protocols for early referral in primary care settings can significantly reduce delays and adverse outcomes. Additionally, leveraging telemedicine to bridge gaps in specialist access can provide real-time ENT guidance, especially in rural or remote areas.

Ultimately, improved patient care demands a strong global research agenda. Standardizing DNSA registries, appraising risk-stratifying tools, and investigating the implementation in different healthcare scenarios are key future perspectives. New technologies (rapid molecular diagnostics, severity prediction models based on biomarkers, and AI-assisted clinical care guidance) are promising but not yet evidence-based endeavors that may improve individualized care in the future. Until DNSA-specific evidence supports these approaches, management should be based on general principles of early diagnosis, selective imaging, interdisciplinary coordination, and timely intervention.

## Figures and Tables

**Table 1:** Geographic and socioeconomic variation in DNSA epidemiology and microbiology.

Region	Incidence Trend	Predominant Etiology	Distinctive Microbiological Features	Reported Mortality	Key Contributing Factors
Northern/Western Europe	Rising [6,32]	Pharyngotonsillar (children), odontogenic (adults)	Streptococci, anaerobes; low MRSA (5–12%) [8,9]	1.6–7.5% [6,32]	Aging population, diabetes prevalence
North America	Rising [13,14,18]	Odontogenic predominant	Higher MRSA prevalence (12–25%) [10]	2–5% [13,21]	Dental care disparities, community MRSA
East Asia (HIC: Japan, Taiwan, South Korea)	Stable to rising [17,23,24]	Similar to Western pattern	Comparable to European profile; Klebsiella in diabetics [16]	1–4% [17]	Aging population, diabetes
South/Southeast Asia (LMIC)	High burden [7,20]	Odontogenic predominant (60–80%)	Klebsiella pneumoniae (diabetics), ESBL-producers [16]	10–20% [7]	Limited dental access, uncontrolled diabetes, delayed presentation
Sub-Saharan Africa	High burden (limited data) [22,46]	Odontogenic predominant	Limited culture data; atypical pathogens in HIV	Higher than HIC [46]	Delayed presentation, HIV co-infection, limited surgical capacity
Latin America	Variable (limited data) [11,28]	Odontogenic predominant	Similar to European/North American pattern	Variable	Urban–rural disparities
Australia/Oceania	Rising [10,30]	Similar to European pattern	MRSA: 12%; ESBL: 8% [10]	1–3%	Indigenous health disparities

## Data Availability

No new data were created or analyzed in this study. Data sharing is not applicable to this article.

## Supplementary Materials

The following supporting information can be downloaded at https://www.mdpi.com/article/10.3390/jpm16030146/s1, Figure S1: Literature screening flow diagram.

## Abbreviations

The following abbreviations are used in this manuscript:

AMR	Antimicrobial resistance
CT	Computed tomography
DNM	Descending necrotizing mediastinitis
DNSA	Deep neck space abscess
ENT	Ear–nose–throat
HIC	High-income countries
ICU	Intensive care unit
LMIC	Low- and middle-income countries
LOS	length of stay
MRI	Magnetic resonance imaging
MRSA	Methicillin-resistant strains
POCUS	Point-of-care ultrasound
UK	United Kingdom
USA	United States of America
WHO	World Health Organization
