# Supplementary figures and images for: Global Burden of Deep Neck Space Abscesses: Epidemiology, Challenges, and Outcomes
Source: J Pers Med. 2026 Mar 3;16(3):146. doi: 10.3390/jpm16030146 (PMC13027759; doi:10.3390/jpm16030146)

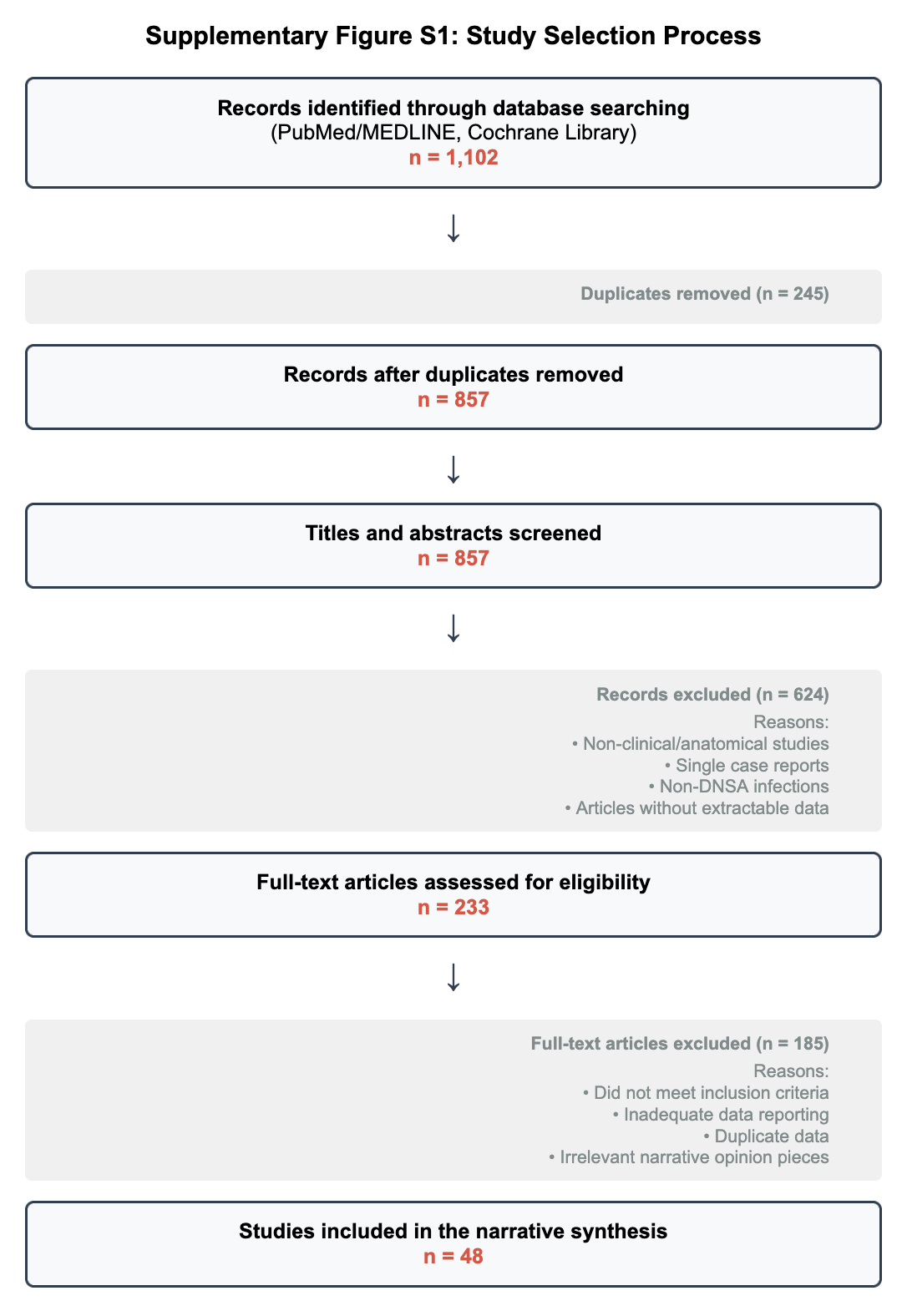

Supplement: Supplementary file 1 [file jpm-16-00146-s001.zip › jpm-4072474-supplementary.png]
